# Clinical Classification of Bone Augmentation Procedure Failures in the Atrophic Anterior Maxillae: Esthetic Consequences and Treatment Options

**DOI:** 10.1155/2019/4386709

**Published:** 2019-02-12

**Authors:** Vittorio Checchi, Roberta Gasparro, Roberto Pistilli, Luigi Canullo, Pietro Felice

**Affiliations:** ^1^Department of Biomedical and Neuromotor Sciences, Unit of Restorative Dentistry, University of Bologna, Via San Vitale 59, 40125 Bologna, Italy; ^2^Department of Neuroscience, Reproductive Sciences, and Dental Science, Unit of Oral Surgery and Implantology, University of Naples, Via Pansini 5, 80131 Naples, Italy; ^3^Oral & Maxillofacial Unit, San Camillo Hospital, 00152 Rome, Italy; ^4^Private Practice, 00198 Rome, Italy; ^5^Department of Biomedical and Neuromotor Sciences, Unit of Periodontology and Implantology, University of Bologna, Via San Vitale 59, 40125 Bologna, Italy

## Abstract

Although the number of complications and failures in bone augmentation procedures is still relatively high, these problems remain poorly documented. Moreover, the literature concerning reconstructive techniques and the treatment of their complications in the anterior areas rarely considers the final esthetic result. The aim of this paper is to propose a new classification of bone augmentation complications in the esthetic area, providing treatment guidelines useful for the management of these cases. Failures of bony regeneration procedures can be mainly divided into partial failures and complete failures. A partial failure can be solved with a corrective surgical intervention: this second surgery can have success or may not be able to provide the desired esthetic result. When the bone reconstructive procedure fails totally, a complete failure occurs and the whole procedure has to be repeated. This new intervention can have success but also this new reconstructive surgery can fail in the same way as the first, causing important damage and a compromise solution that will hardly be acceptable from an esthetic point of view. Bone augmentation techniques are not completely predictable and are not always able to guarantee the expected result, especially in the atrophic anterior maxilla. Complications and failures can often occur and this possibility must always be clearly explained to those patients with high esthetic demands and expectations.

## 1. Introduction

The rehabilitation of the partially edentulous maxillae with implant-supported prosthesis is a frequent procedure, with reliable long-term results [1]. However, due to periodontitis, trauma, agenesis, and/or tooth extractions, alveolar bony defects and anatomical modifications in bone height and width can occur. In these conditions, the placement of prosthetically-oriented dental implants may be difficult [2].

The ideal approach is always to augment the bone vertically and horizontally in the most predictable and successful way possible.

Current reconstructive approaches include several techniques, with different success rates, such as interpositional grafts [3, 4], onlay block bone grafting [5–7], the ridge split technique/ridge expansion [8], guided bone regeneration (GBR) [9, 10], and distraction osteogenesis [9]. In the anterior maxilla, the first therapeutic option to better provide functional and esthetic results is alveolar ridge augmentation [2, 6]. Although, conventionally, autogenous bone is considered the gold standard for alveolar ridge augmentation [2, 3, 6, 11, 12], the surgery in the donor site and obviously limited amount of the harvested bone can create clinical obstacles [12–14]. Moreover, the resorption of grafted autogenous bone is a common and unwanted complication, which compromises the long-term stability [12–14].

Many different bone substitutes have been developed to be used as scaffolds to promote cell migration, proliferation, and differentiation [15, 16]. Bone allografts and xenogenic biomaterials have been widely used for vertical and horizontal reconstructions of the alveolar ridges, as an alternative to autogenous bone grafts [3, 17, 18].

Various reconstructive techniques have been well described in the literature [2, 3, 6–11], but although the number of complications and failures is still quite high [11], they remain relatively little documented. In different percentages [1], complications can occur during the surgery, in the early or in the late healing process, and may be situated at the recipient or donor site, when autogenous bone is used [19]. To the best of our knowledge, there is no study in the literature that has investigated and has been able to quantify the incidence of complete failures in reconstructive procedures in the anterior maxilla.

Moreover, the literature concerning bone augmentation procedures and the treatment of their complications in the anterior areas rarely considers the final esthetic result.

The aim of this paper is to propose a new classification of bone augmentation complications in the esthetic area, with the intention of providing treatment guidelines useful for the management of these clinical problems.

## 2. Classification and Treatment Options

### 2.1. Classification of Bone Augmentation Complications in the Esthetic Area

**Figure 1:**
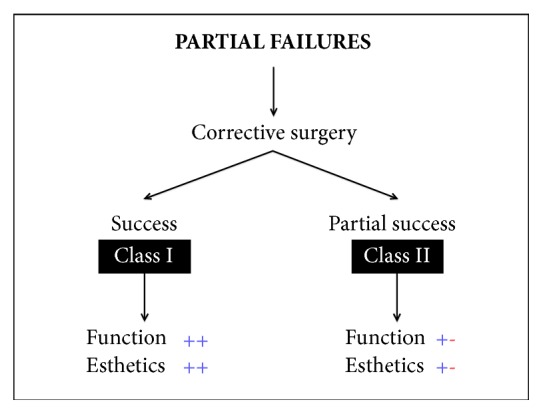


**Figure 2:**
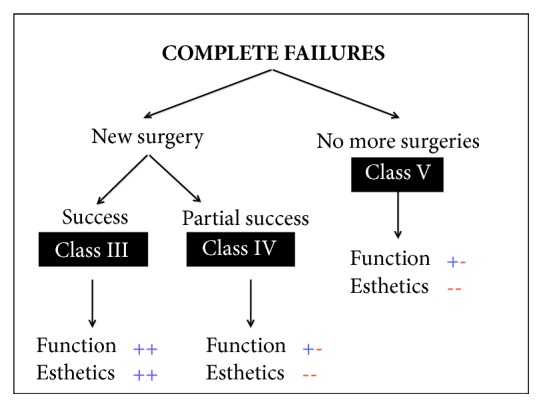


Failures of bony regeneration procedures can be mainly divided into Partial Failures and Complete Failures (Figures 1 and 2).

A failure occurs when the expected result has not been achieved, due to events which may normally cause a deviation from the usual course of surgical progress. Failures may be the result of factors inherent to the treated condition, related to other conditions, or due to intraoperative or postoperative complications [20].

Partial failure occurs when the surgical procedure does not lead to the expected results but there is still a possibility of achieving the initial goal with one or more surgical corrections.

A complete failure instead occurs when the whole procedure fails and the surgery has to be performed again, often with lower amount and poorer quality hard and soft tissues than those used in the initial presurgical condition.

- A* Partial Failure* can be solved with a corrective surgical intervention.
  - In the best case, this second surgery can have success, leading to a good functional and esthetic result (*Class I*).
  - However, the second corrective surgery is often not able to provide the desired esthetic solution. In these cases we can experience a loss of interdental papillae or the requirement for a prosthesis with artificial soft tissues (*Class II*).
- If the bone reconstructive procedure fails totally, a* Complete Failure* occurs and the whole procedure has to be performed again.The result of this new intervention is as unpredictable as the initial surgery.
  - It can have success (*Class III*) but, unfortunately, also this new reconstructive surgery can fail in the same way as the first intervention.
  - In this case we can have important damage and, in order to guarantee at least an acceptable function to the patient, we are forced to accept a compromise that will be hardly acceptable from an esthetic point of view (*Class IV*).

Another possibility in the case of a Complete Failure of a bone regeneration procedure is to decide not to perform the same procedure again but to try to limit the damage with nonsurgical corrections. This decision could be influenced by the preferences of the patient who may wish to obtain a functional result without undergoing new surgical procedures. This solution will be a compromise solution from both a functional and an esthetic point of view (**Class V**).

### 2.2. Treatment Option for a Class 1 Complication

**Figure 3:**
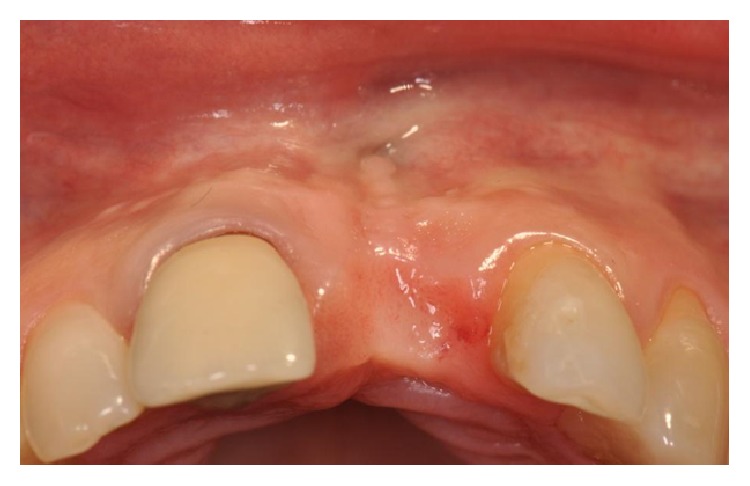
Clinical view showing a lack of the upper left central incisor.

This case shows the loss of the upper left central incisor due to endodontic failure (Figure 3).

**Figure 4:**
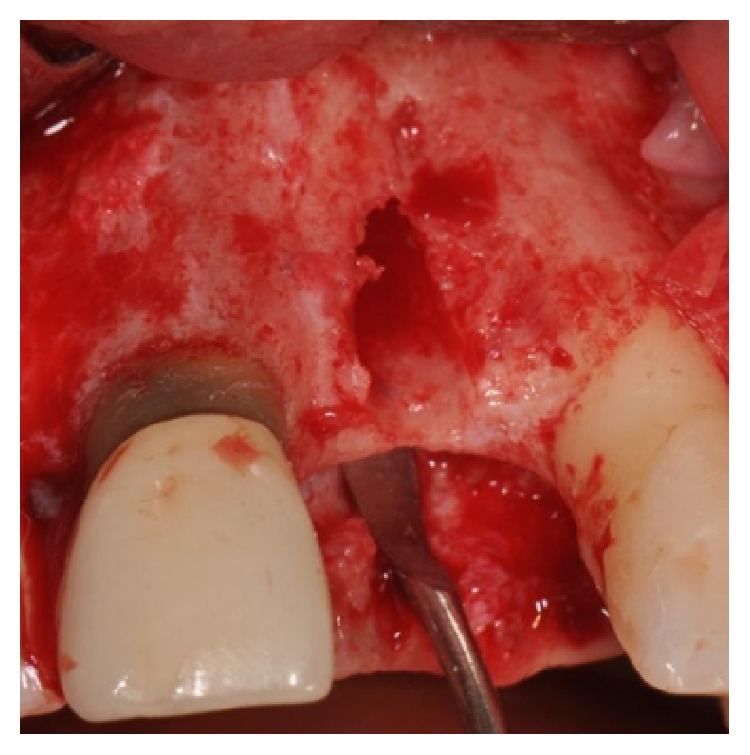
Surgical view of the socket; the whole buccal bony wall is missing.

Four months after the extraction, a full thickness flap was raised; the socket was still present, but the whole buccal bony wall was missing (Figure 4).

**Figure 5:**
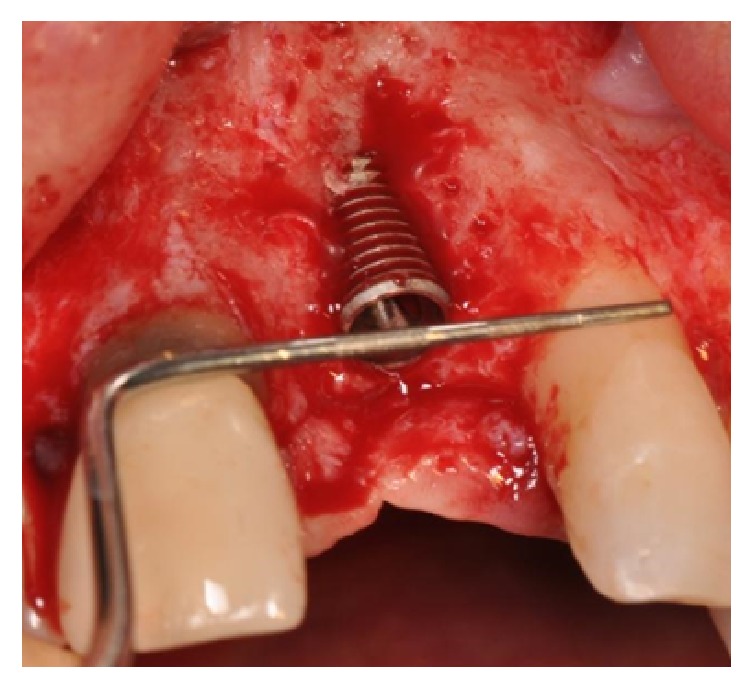
One implant is placed in the socket with all its coronal buccal part exposed.

One implant (Megagen AnyRidge, Megagen, Korea) was placed in the socket with all its coronal buccal part exposed (Figure 5).

**Figure 6:**
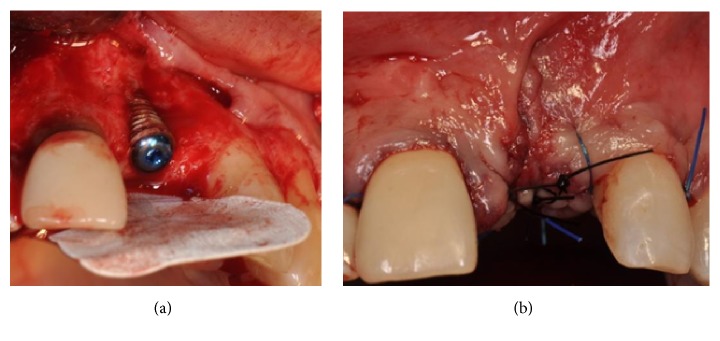
(a-b) GBR procedure performed using a microtextured titanium-reinforced PTFE membrane, fixed by one pin.

Using a microtextured titanium-reinforced PTFE membrane (Cytoplast® Ti-250, De Ore®, Italy), fixed by one pin (Pro-fix® Membrane Fixation System, Osteogenics Biomedical, Texas, USA), a GBR procedure was performed in order to gain the amount of bone necessary to reestablish a correct profile (Figures 6(a) and 6(b)).

**Figure 7:**
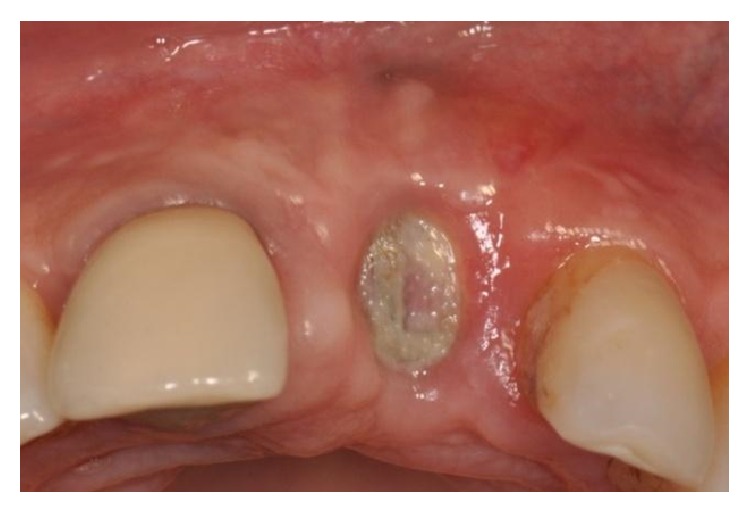
Membrane exposure after three weeks of healing.

After three weeks of healing, the membrane was exposed in its coronal part (Figure 7).

**Figure 8:**
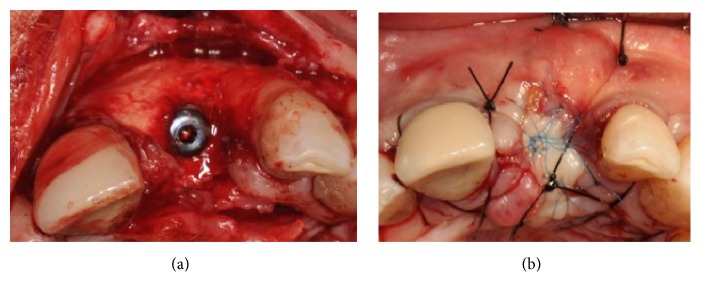
(a-b) Connective tissue graft harvested from the left palate in order to close the flap fenestration.

The area was treated with applications of chlorhexidine gel (Corsodyl gel, GlaxoSmithKline, UK) twice a day for seven days. After this period, a full thickness flap was raised and a connective tissue graft was harvested from the left palate and placed coronally to the implant in order to close the flap fenestration (Figures 8(a) and 8(b)).

**Figure 9:**
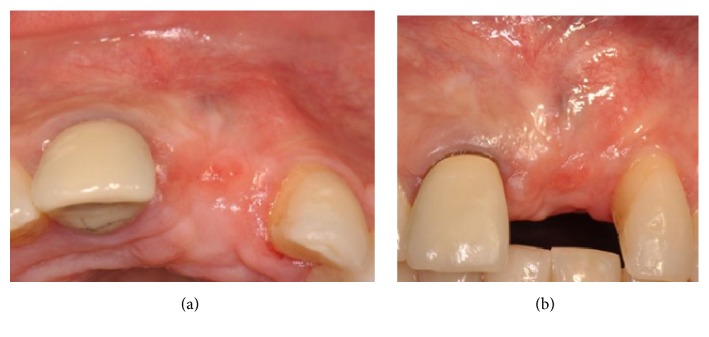
(a-b) Healing of the soft tissues with a reduced thickness of buccal profile.

After 45 days of healing, the soft tissues were healthy and keratinized tissue was present, but the buccal profile was still not sufficiently thick (Figures 9(a) and 9(b)).

**Figure 10:**
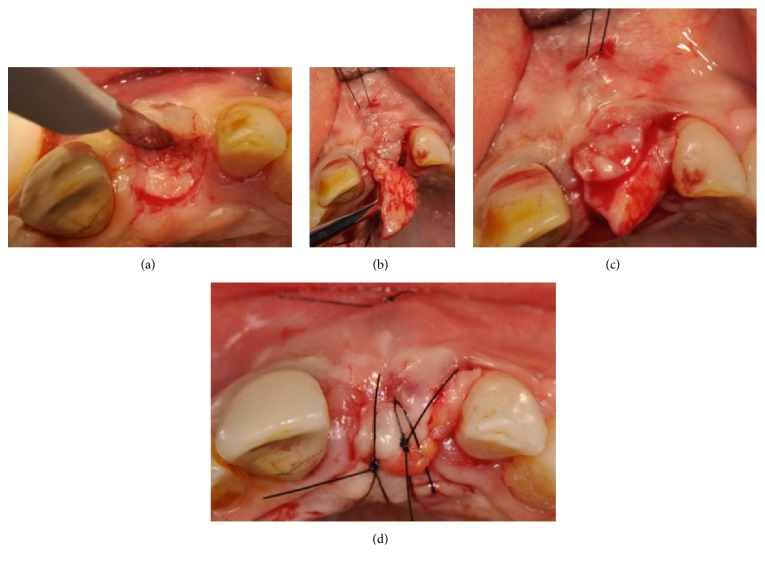
(a-b-c-d) A partial thickness flap is created to house a second connective tissue graft, harvested from the right palate and placed buccally.

With a crestal incision and no vertical release cuts, a partial thickness flap was created that was able to house a second connective tissue graft, harvested from the right palate and placed buccally (Figures 10(a), 10(b), 10(c), and 10(d)).

**Figure 11:**
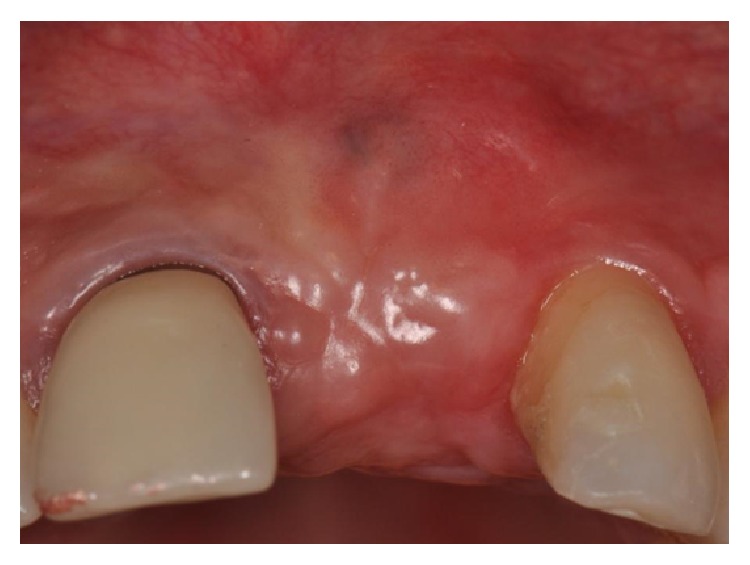
Clinical view of the edentulous area: reduced soft tissue volume at the level of the distal papilla.

After two months of healing, the edentulous area of the upper central left incisor finally achieved a good buccal profile but was still lacking sufficient soft tissue volume in relation to the distal papilla (Figure 11).

**Figure 12:**
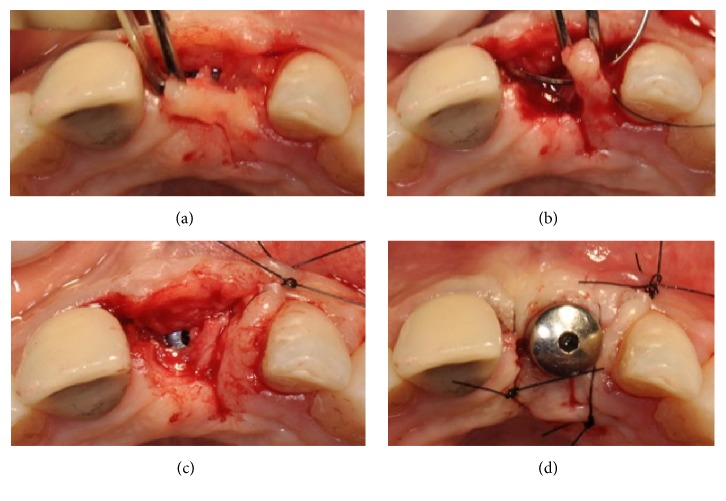
(a-b) A narrow part of the palatal flap is cut to half-thickness and rotated distally against the lateral upper left incisor. (c-d) The papilla mesial to the lateral incisor is disepithelized and the microflap is sutured over the original papilla.

To solve this final problem, which may have prejudiced the final esthetic result, a laterally translated microflap was created (Figures 12(a), 12(b), 12(c), and 12(d)).

A narrow part of the palatal flap was cut to half-thickness (Figure 12(a)) and rotated distally to the lateral upper left incisor (Figure 12(b)). The papilla mesial to the lateral incisor was disepithelized and the microflap was sutured over the original papilla (Figures 12(c) and 12(d)).

**Figure 13:**
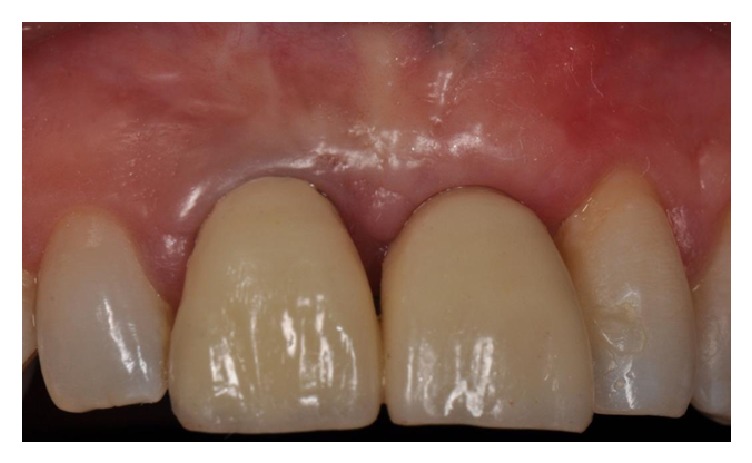
Provisional crown delivered with a slight compression on the newly formed papilla, after three weeks of healing.

After three weeks of healing a provisional crown was inserted with only a slight compression on the newly formed papilla (Figure 13).

**Figure 14:**
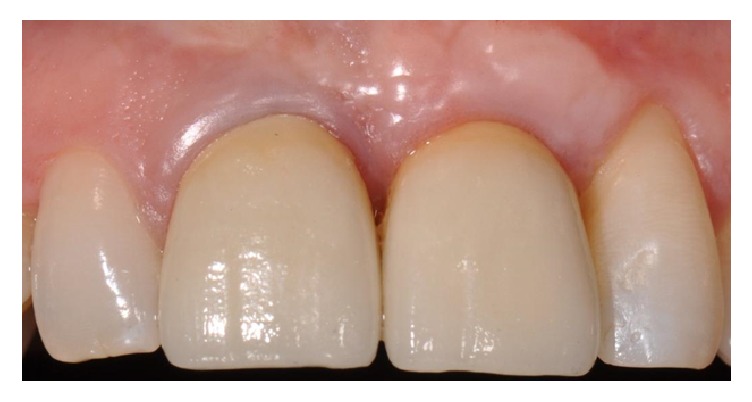
Definitive ceramic crown is placed after two months.

Finally, two months later, a definitive ceramic crown was placed, surrounded by healthy soft tissues, with a good amount of buccal keratinized tissues and the presence of the papilla between the central and lateral left incisors (Figure 14).

### 2.3. Treatment Option for a Class 2 Complication

**Figure 15:**
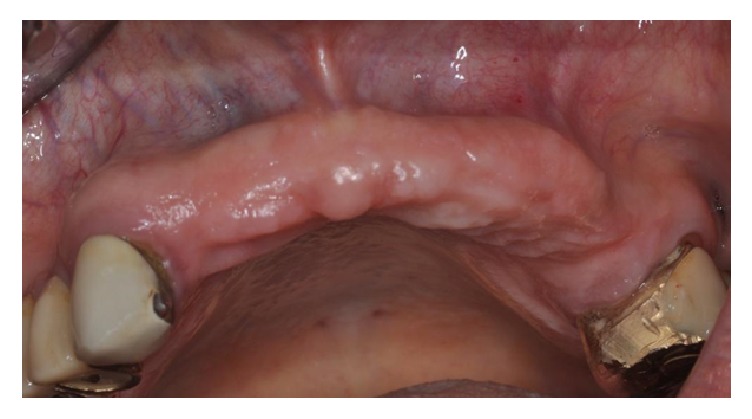
Clinical view of the upper anterior maxilla showing an absence of all the anterior teeth combined with a lack of adequate buccal thickness.

This case shows an absence of all the anterior superior teeth combined with a lack of adequate buccal thickness (Figure 15).

In order to obtain an implant-supported rehabilitation, implant placement with contextual bone regeneration was planned.

**Figure 16:**
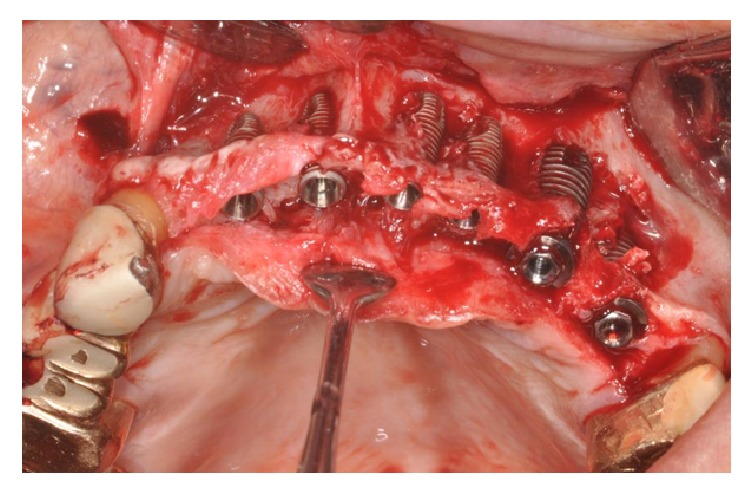
Placement of six dental implants with the bone surrounding only their coronal part.

Taking advantage of the presence of a thick amount of crestal cortical bone, six dental implants (ExFeel, Megagen®, Korea) were placed with the bone surrounding only their coronal part (Figure 16).

**Figure 17:**
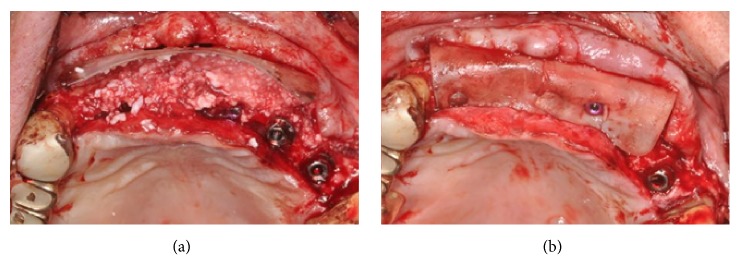
(a-b) Granules of biomaterial are placed between the implants and in excess on the buccal side, covered by a semi-rigid resorbable polylactic acid membrane and fixed with implant cover screws and resorbable tacks.

To cover the buccal aspect of the implants and in order to compensate for the lack of regenerate bone, granules of biomaterial (Mp3, Osteobiol, Italy) were placed between the implants, in excess on the buccal side, covered by a semi-rigid resorbable polylactic acid membrane (GTR Biodegradable membrane, Inion®, Finland), fixed with both implant cover screws and resorbable tacks (GTR Tack, Inion®, Finland) (Figures 17(a) and 17(b)).

**Figure 18:**
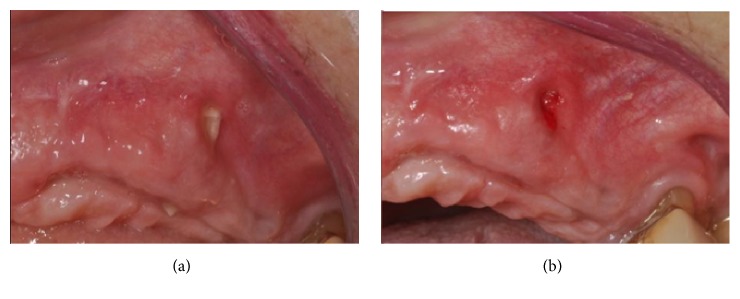
(a-b) Partial exposure of an angle of the membrane, which occurred after four months and was treated with a little plastic.

The flaps were sutured and primary closure, with no tension, was obtained. The healing was uneventful, except for a partial exposure of an angle of the membrane, which occurred after four months, and was immediately treated and resolved with a little plastic (Figures 18(a) and 18(b)).

**Figure 19:**
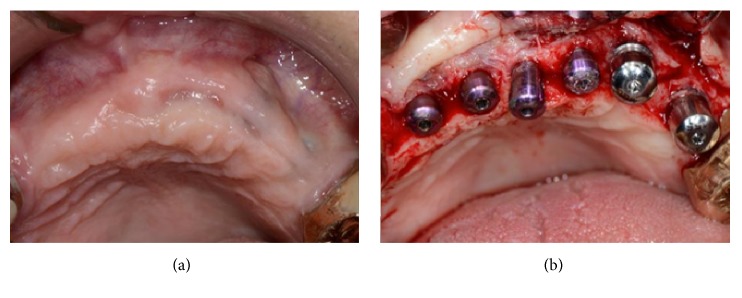
(a-b) Eight months after the implant therapy, the implants appeared stable and well osseointegrated, but the buccal profile was still not sufficiently thick.

Eight months after the implant placement, the healing screw stage was completed. The implants appeared stable and well osseointegrated, but the buccal profile was still not sufficiently thick to provide a good esthetic result (Figures 19(a) and 19(b)).

**Figure 20:**
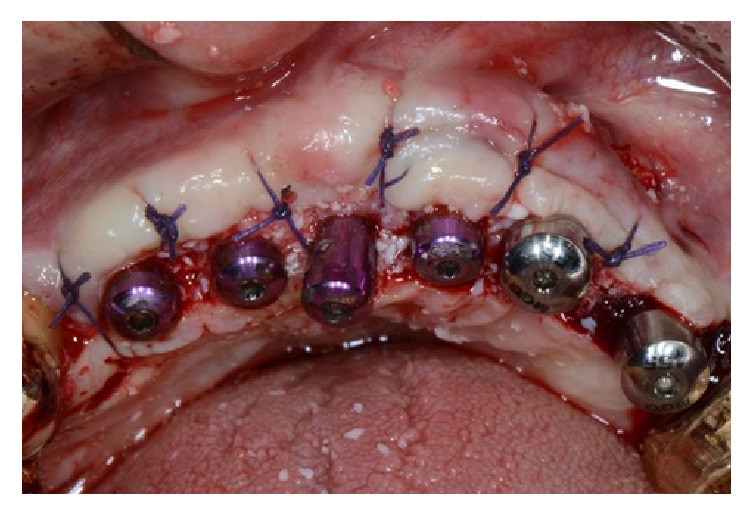
Grafting material is placed buccally under the flap to improve the thickness of the profile and the flaps are left to heal for a second intention.

Therefore, the same grafting material was placed buccally under the flap to improve the thickness of the profile and the flaps were left to heal for a second in order to obtain a greater amount of keratinized gingiva (Figure 20).

**Figure 21:**
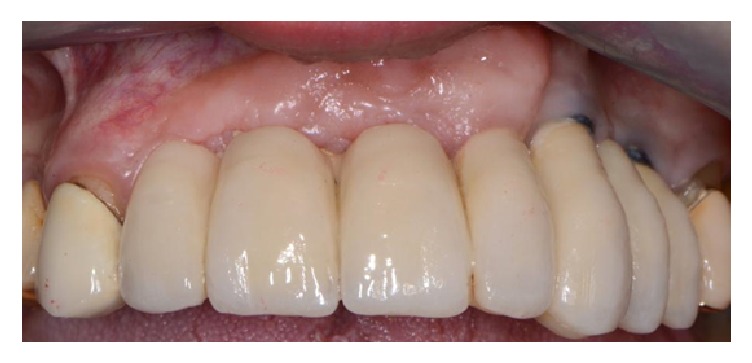
Final prosthesis delivered to the patient.

Despite these attempts to increase the buccal profile of the maxilla, the final result was satisfactory only from a functional point of view since no good esthetic result was obtained (Figure 21).

### 2.4. Treatment Option for a Class 3 Complication

**Figure 22:**
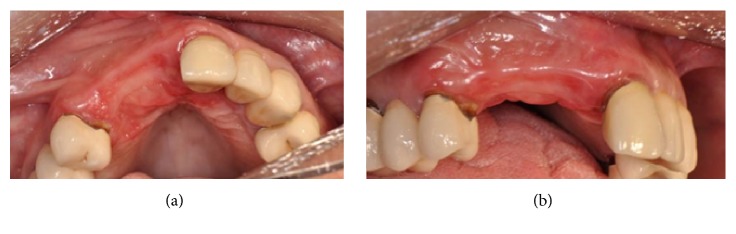
(a-b) Clinical frontal and occlusal views of the atrophy in the upper right frontal area.

This patient presented a loss of teeth #11, 12, and 13 due to severe periodontal disease (Figures 22(a) and 22(b)).

In order to rehabilitate both the teeth and alveolar bone, implant placement with a concurrent GBR procedure was planned.

**Figure 23:**
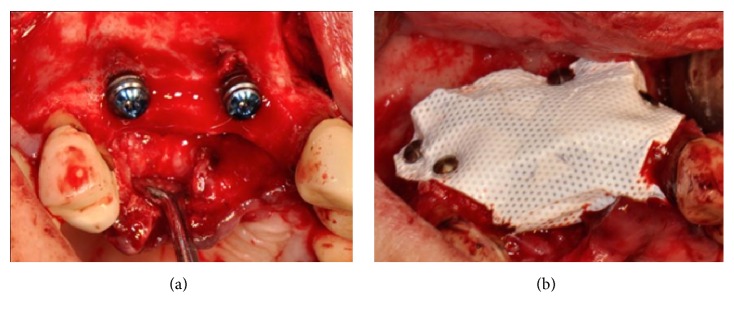
(a) Two implants placed supracrestally in sites #13 and #11. (b) The lack of crestal and buccal bone volume is compensated for with a 1:1 mixture of particles of bovine derived xenograft and autogenous bone harvested from the premaxilla and the palate with bone scrapers. The bone graft is covered with a microtextured titanium-reinforced PTFE membrane and fixed by pins and miniscrews.

Implants (AnyRidge, Megagen, Korea) were placed supracrestally in sites #13 and #11 and the lack of crestal and buccal bone volume was compensated for with a 1:1 mixture of particles of bovine derived xenograft (Bio-Oss®, Geistlich, Switzerland) and autogenous bone harvested from the premaxilla and the palate with bone scrapers. The bone graft was then covered with a microtextured titanium-reinforced PTFE membrane (Cytoplast® Ti-250, De Ore®, Italy) and fixed by pins and miniscrews (Pro-fix® Membrane Fixation System, Osteogenics Biomedical, Texas, USA) (Figures 23(a) and 23(b)).

**Figure 24:**
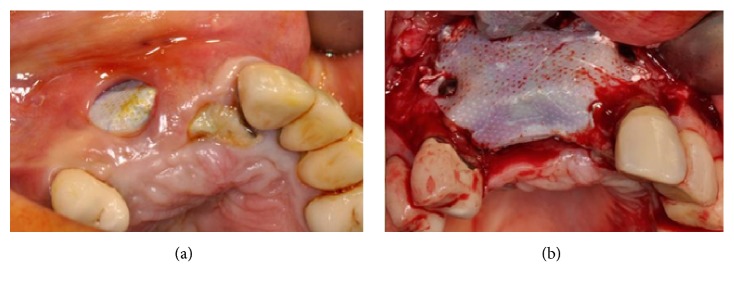
(a) Exposure of the membrane on the buccal aspect of the area after five months of healing. (b) Clinical situation after reopening the flap; the membrane, pins, and screws were removed.

After five months of healing, an exposure of the membrane on the buccal aspect of the area was detected (Figure 24(a)). Antibiotic therapy (Augmentin® 1 gr every eight hours for seven days) was administered starting three days before the surgery.

The surgical site was reopened with a crestal and sulcular incision around teeth #14 and #21, with two vertical releasing incisions. The flap was raised to full-thickness and all pins and screws were removed (Figure 24(b)). The underlying tissue revealed a good solidity except in the buccal area around implant #13. In this area, the tissue was removed causing a 1.5 mm exposure of the implant neck. Crestally, a modest amount of phlogistic soft tissue was also removed, with washing with a saline solution.

**Figure 25:**
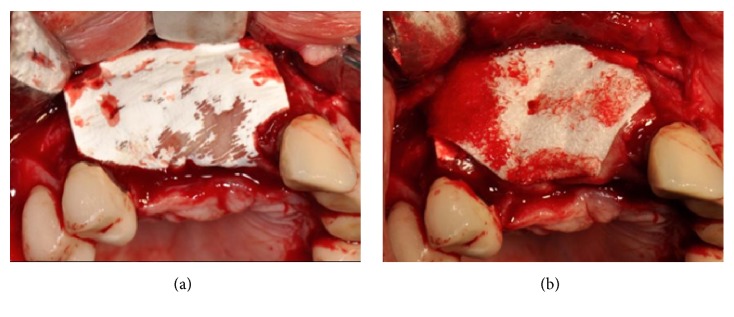
(a-b) A resorbable membrane is placed, covered by a collagen sponge, in order to create a double-layered barrier protecting the bony area that required regeneration.

A resorbable membrane (BioGide, Geistlich, Switzerland) was then placed covered by a collagen sponge (Medicipio, De Ore, Italy), in order to create a double-layered barrier protecting the regenerated bony area (Figures 25(a) and 25(b)).

**Figure 26:**
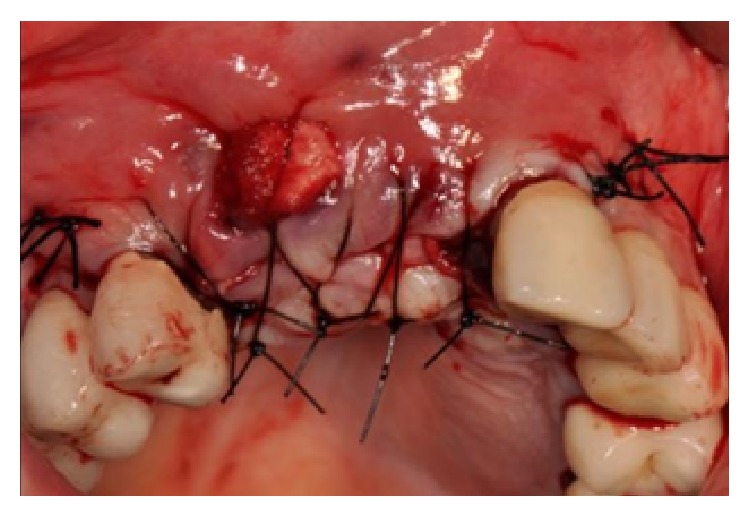
Closure of the flap is obtained after suturing with a deliberate lack of first intention; the fenestrated buccal area is left to heal for a second intention over the collagen sponge.

Closure of the flap was obtained after suturing and the fenestrated buccal area was left to heal for a second intention over the collagen sponge (Figure 26).

The decision not to attempt to obtain a first intention closure was related to the lack of soft tissues available and to the need for keratinized tissue in the crestal area. All the regenerative potentiality of the underlying tissue protected by the barrier was exploited.

**Figure 27:**
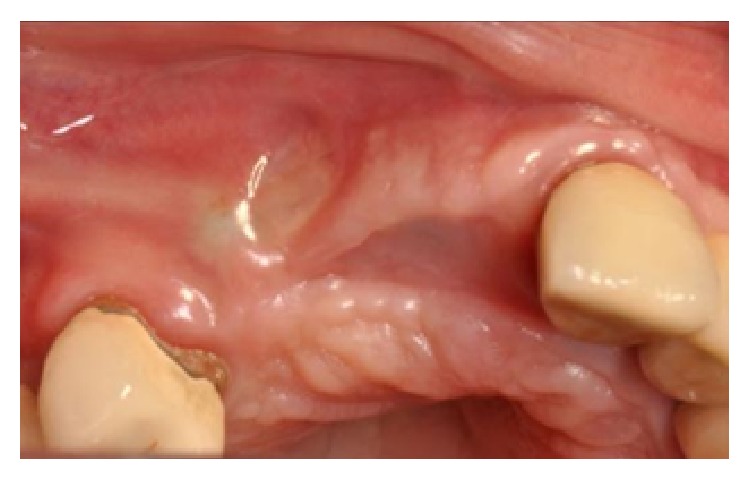
Clinical view of the closure of the fenestration after one month of healing.

After one month of healing, the fenestration of the mucosa was no longer present and all the area was completely reepithelized again (Figure 27).

**Figure 28:**
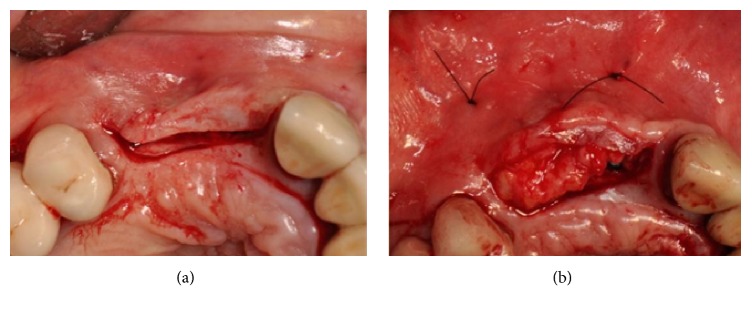
(a-b) A connective tissue graft is harvested from the palatal left premolar area and placed buccally under the epithelium to compensate for the lack of volume of the buccal alveolar crest.

In order to finalize the procedure, a connective tissue graft was harvested from the palatal left premolar area and placed buccally under the epithelium to compensate for the lack of volume of the buccal alveolar crest (Figures 28(a) and 28(b)).

**Figure 29:**
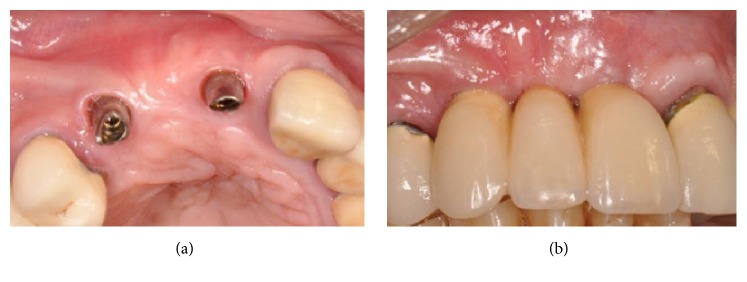
(a-b) Final prosthesis delivered after four months.

After five months of healing of the soft tissues, healing screws were placed on the two implants and a provisional restoration was achieved. The final prosthesis (Figures 29(a) and 29(b)) was performed after another four months, in a healthy and keratinized gingiva but with a clear lack of interdental papillae.

### 2.5. Treatment Option for a Class 4 Complication

**Figure 30:**
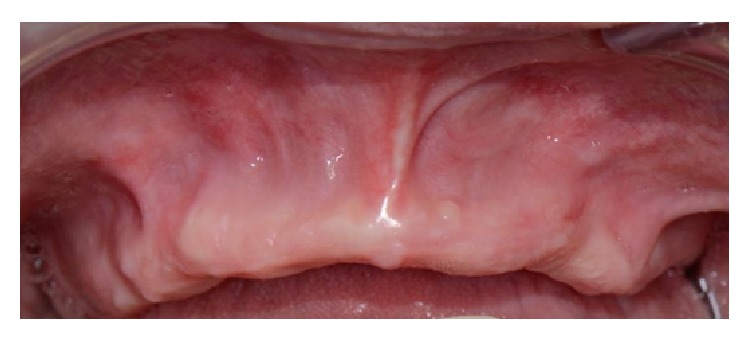
Clinical view of the severe bone resorption of the upper maxilla.

This case presents a severe bone resorption of the upper maxilla due to early tooth loss (Figure 30). The treatment plan considered an onlay block graft procedure followed by implant placement.

**Figure 31:**
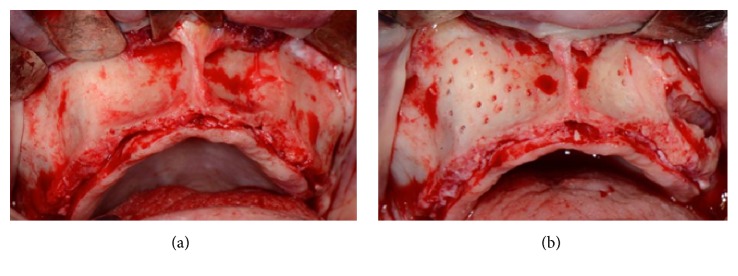
(a-b) Surgical view of the resorbed area and bone refreshing.

**Figure 32:**
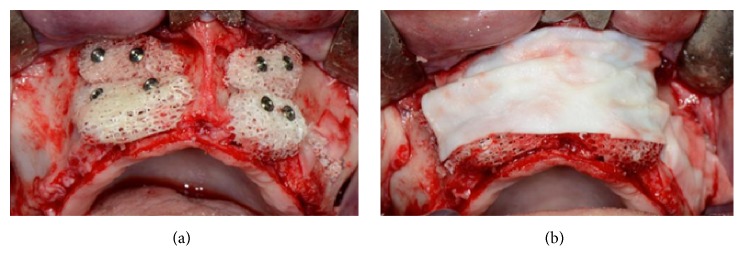
(a) Placement and adaptation of four cancellous bone blocks to the recipient site, fixed to the basal bone by miniscrews. (b) The whole grafted area is covered by a resorbable membrane.

After a full thickness raising of the flap and bone refreshing (Figures 31(a) and 31(b)), four cancellous bone blocks (OX Block, Bioteck®, Italy) were adapted to the recipient site and fixed to the basal bone through miniscrews (Micro System, Martin GmbH, Germany) (Figure 32(a)). The whole grafted area was covered by a resorbable membrane (OX Membrane, Bioteck, Italy) (Figure 32(b)).

At the same time, the left sinus was elevated and grafted with the same graft material, in particulate form, and covered with the same membrane. The flaps were sutured with no tension and primary closure was obtained.

**Figure 33:**
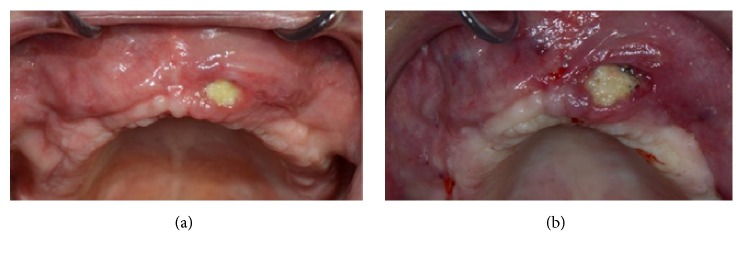
(a) Dehiscence of the flap after six months of healing. (b) Clinical view of the dehiscence: a part of one of the blocks is exposed.

After six months of healing, a dehiscence of the flap was reported and, two weeks later, part of one block was exposed (Figures 33(a) and 33(b)).

**Figure 34:**
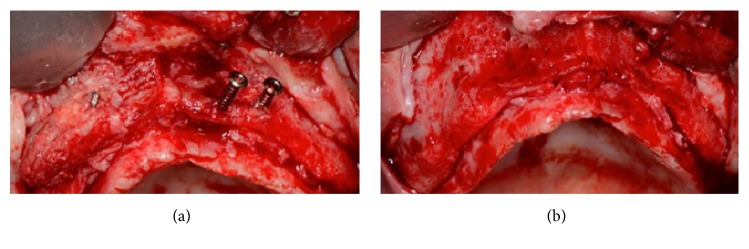
(a-b) During surgical reentry, the grafted blocks appear to be not integrated and the presence of infection and fibrous granulation tissue is evident. The blocks and screws were removed and the whole area was cleaned.

The flaps were raised again and, from a clinical point of view, the grafted blocks appeared not integrated with the presence of infection and fibrous granulation tissue. The blocks and screws were removed and the whole area was cleaned (Figures 34(a) and 34(b)).

**Figure 35:**
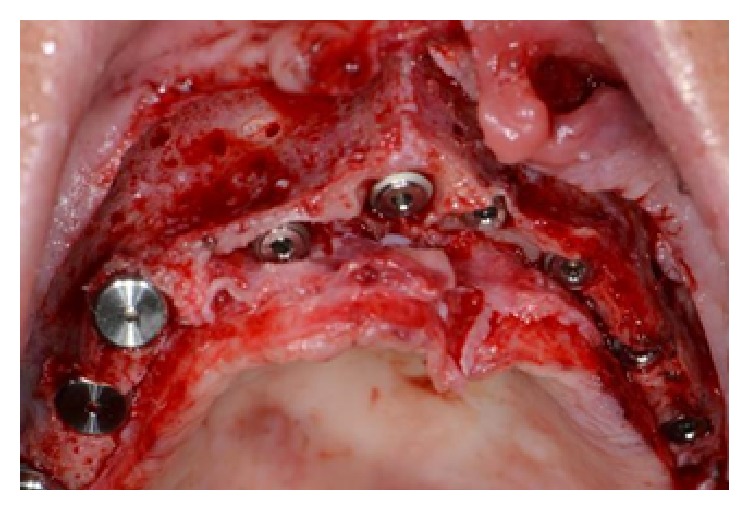
Placement of ten implants in the residual patient native bone.

The surgical area presented the same lack of width as had been the case in the presurgical situation. Despite this lack of bone width, it was decided to place eleven implants, five of them in the anterior region, positioned in the residual patient native bone (Figure 35).

**Figure 36:**
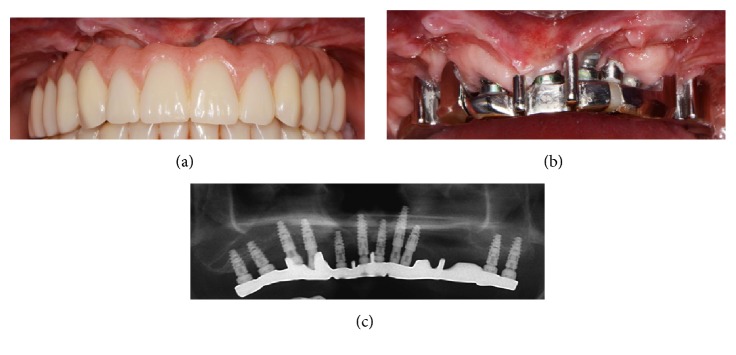
(a-b) The implant-retained combined fixed-removable denture delivered to the patient four months after implant therapy. (c) Panoramic X-ray of the final prosthesis.

After four months of healing, the implants were osseointegrated and an implant-retained combined fixed-removable denture was achieved (Figures 36(a), 36(b), and 36(c)).

The final result may be considered acceptable from a functional point of view, but the failure of the onlay grafting procedure caused an inadequate thickness of both soft and hard tissues.

## 3. Discussion

In our classification, a Class I partial failure signifies a successful corrective surgical intervention that leads to a good functional and esthetic result. In our case, the problems relating to soft tissue fenestration, lack of buccal thickness, and lack of interdental papillae were resolved with mucogingival corrective procedures that led to good final results.

A Class II partial failure is characterized by corrective surgery that is not able to provide the desired esthetic result. In the case presented, the initial bone grafting procedure favored the implant integration but was not able to provide an adequate buccal volume. Consequently, new bone graft particles were added to resolve this issue. Despite these corrective procedures, the final result showed a good prosthetic rehabilitation but with a poor esthetic appearance.

Class III indicates the group of complete failures, cases which require that the whole procedure has to be performed again to achieve a final success. In our case, the bone regeneration procedure had to be repeated. The final result, however, even after a connective tissue graft, was successful from both an esthetic and a functional point of view. 

In the case of a Class IV total failure, the second reconstructive surgery fails in the same way as the first intervention, with important tissue damage and the acceptance of a compromise solution which is hardly acceptable from an esthetic point of view. In the case presented, bone block graft exposure caused an infection and a failure in the augmentation procedure. The final solution is certainly not acceptable from an esthetic point of view and is only barely acceptable from a functional point of view.

Only a few research groups have tried to create a classification of the complications of bone reconstructive procedures.

Merli et al. evaluated the complications of two different techniques for vertical bone augmentation, without making any distinction between the mandible and maxilla [21]. The authors classified GBR complications as major or minor. Major complications were those that caused a failure of the graft, such as an infection in the dehiscence of the soft tissues or abscesses. These failures needed additional surgery and systemic antibiotics. Minor complications instead were those that did not result in a complete loss of the graft, such as a dehiscence of the soft tissues with no treatment required or only applications of chlorhexidine and/or systemic antibiotics [21].

In a clinical paper, Verardi and Simion suggested a division into two classes, depending on the treatment options for nonresorbable membrane exposure in GBR procedures. A Class I complication was defined as a small soft tissue fenestration (≤ 3 mm), while a Class II was defined as a wider opening (> 3 mm) [22].

Fontana et al. proposed a classification of the complications in GBR with the intention of providing guidelines for the management of these clinical situations [23]. Since there was no information reported in the literature regarding the treatment modalities of these complications, their clinical approaches were not evidence-based but only recommendations based on their clinical experience. The authors identified two major categories of complication: healing complications and surgical complications. The first group was classified as follows: Class I (small membrane exposure without purulent exudate), Class II (large membrane exposure without purulent exudate), Class III (membrane exposure with purulent exudate), and Class IV (abscess formation without membrane exposure) (Table 1) [23].

Exposures of Class I reveal a fenestration smaller than 3 mm with no purulent exudate. The authors suggest not removing immediately the membrane but rather leaving it in place for a maximum period of one month. However, topical applications of 0.2% chlorhexidine gel twice a day to reduce plaque formation and weekly patient follow-up appointments are mandatory. In some detailed cases, a small soft tissue fenestration could instead be treated with the removal of the exposed portion of the membrane and the closure of the dehiscence with a connective tissue graft or with a suture.

Class II concerns an exposure larger than 3 mm with no purulent exudate. In these cases, the membrane must be removed immediately to avoid any possible interference with the regeneration process. Luckily, in many cases, the underlying graft is not compromised. Therefore, after membrane removal, the grafted area has to be left in place and submerged for at least four months.

In Class III, in a case of membrane exposure associated with purulent exudation, the membrane has to be removed immediately, and the underlying infected graft and inflammatory tissue must be curetted and removed. If this complication occurs within the first month after the grafting procedure, the chance of preserving even a part of the graft is minimal. A period of at least two months of undisturbed healing processes is essential before performing a second regenerative procedure.

Class IV represents cases with the most severe complications: the formation of an abscess in the regenerated area without any exposure of the membrane. This often happens within the first month after the surgery and is correlated with a bacterial contamination of the graft and the membrane. Immediate membrane removal is mandatory, associated with a complete curettage of the graft, local antibiotic washes, and the oral administration of systemic antibiotics. In the case of membrane removal delay or the presence of severe infection, partial resorption also of the native basal bone is expected.

Moreover, the authors classified surgical complications in A (flap damage), B (neurological complications), and C (vascular complications) (Table 1) [17]. The release of buccal and lingual flaps is a critical phase for the management of the soft tissues in regenerative procedures. An improper handling of the flaps can lead to their damage and neurological or vascular complications.

Flap damage (A) could be a consequence of an excessively deep periosteum incision. An excessive thinning of the flap or flap perforation may cause a reduction of vascular supplies and, consequently, a necrosis of the soft tissues. This necrosis often causes the failure of any regenerative procedure.

In the maxillae, neurological complications (B) are represented by lesions to the infraorbital nerve. Nerve damage is the consequence of a direct trauma to the nerve fibers from the surgical instruments, which can cause temporary or permanent sensory effects (anesthesia, paresthesia, or dysesthesia).

Vascular complications (C) are considered in terms of edema and hemorrhaging in the sublingual space [23].

Jensen et al. recently defined lesions as early if they appear within twenty-one days postoperatively, or late if they appear after that period. They registered the complications as follows: (1) soft tissue dehiscence (separation of the suture line with an exposure of the barrier membrane and/or the grafting material); (2) infection (pain, swelling, redness, fever, and/or purulent discharge that require(s) additional antibiotic treatment); (3) additional augmentation procedures needed at the time of the implant placement (to obtain sufficient implant stability and/or to create an optimal esthetic result after the loss of a graft or an inadequate primary augmentation) [19].

A case report by Mau et al. proposed a GBR performed four months after a previous failed regeneration, probably due to a foreign body reaction to the graft material [24].

In the onlay block graft technique, dehiscence and bone exposure are complications related to bone block graft failure [25]. The soft tissue collapse with block exposure is linked to the slow revascularization of the graft which causes a delayed healing [26].

A systematic review of the clinical outcomes of vertical bone augmentation was not effective in providing any information about the complications caused by the morbidity of extraoral bone grafts for onlay bone grafting [2]. All the complications reported in this review concerned the effects of bone harvesting from intraoral sites [2].

Jensen et al. reported twenty-three cases of bone defects in which a staged horizontal ridge augmentation was performed, twelve treated with autogenous bone blocks and eleven with GBR. An additional bone augmentation procedure was necessary before implant therapy in 37% of cases. Seven cases of dehiscence occurred (two early and five late) and infections developed at three sites (two early and one late) [19].

In GBR procedures, the most common complication is membrane exposure [23]. When this happens, the amount of regenerating tissue under the barrier is prejudiced [27–29]. The consequences of wound dehiscence and/or membrane exposure can range from membrane removal and incomplete bone growth to much more serious effects, such as complete treatment failure and the prevention of implant placement [27–29].

In the previously mentioned study, 240 GBR procedures were performed in 171 patients. In five cases, an additional bone augmentation was necessary; five infections (one early and four late) and four cases of dehiscence were recorded (two early and two late) [19].

In the systematic review by Rocchietta et al., a broad range of complications (0-45.5%) was reported in all the studies included. However, only a few of these related to atrophies of the upper maxilla and none of them considered the anterior sector [2].

In the case of a failure of the bone augmentation procedures in the upper maxilla, there are different and more severe consequences than in the mandible. This happens because very often in the mandible there is a lower commitment towards achieving not only a good functional result but also an acceptable esthetic result [17]. However, only a few failed bone augmentation procedures can be solved perfectly, from both a functional and an esthetic point of view. In case of a failure of bone regeneration in the posterior mandible, it is possible to remedy the problem by using short-length implants. This solution does not satisfy the patient in terms of the esthetic result but can guarantee an acceptable masticatory function. In the upper maxilla instead, particularly in the anterior region, this result is not acceptable.

When the treatment plan considers an implant-supported prosthetic rehabilitation, one of the main goals is to obtain harmonious soft tissue profiles [30, 31].

One of the most common esthetic problems is the presence of excessively long clinical crowns. Crown length is the distance from the incisal extremity to the most apical part of the parabola of the gingival margin. If the amount of soft tissues is deficient or in the case of an insufficient soft tissue augmentation, the clinical crown appears too long, demonstrating an evident esthetic failure [32].

In other cases, the loss of soft tissues in the interproximal area makes the creation of a papilla around the implant site impossible. This loss can reveal itself from the beginning of the treatment plan or it may appear as an iatrogenic factor, following an incorrect surgical technique. Since the reconstruction of the papilla between the implants is extremely difficult, the treatment plan and the surgical technique have to be very accurate and precise [33].

Another esthetic failure is a deficiency of the buccal volume. When the volume of the tissues is inadequate on the buccal aspect, it appears as a concavity in relation to the adjacent tissue levels, which causes a characteristic dark shadow. The presence of adequate vertical tissue volumes is not sufficient to prevent this shadowing effect which can only be avoided with the correct amount of tissue volume on the buccal side [7].

All these kinds of esthetic failure can definitely be associated with an incorrect surgical procedure or an insufficient bone and/or soft tissue augmentation. However, it has to be highlighted that they can also result from a surgical complication or a complete failure of the regeneration.

## 4. Conclusions

Complications and failures can often occur after bone augmentation procedures. These techniques are not completely predictable and are not always able to guarantee the expected result, especially in the atrophic anterior maxilla.

Due to the unpredictability of these kinds of procedures, it is necessary to discuss the possibility of complications with the patient from the beginning of the therapy, especially with those patients with high esthetic demands and expectations.

## Figures and Tables

**Table 1:** 

Authors	Surgical procedure	Complication classification	Description of the complication
Merli et al. 2007	GBR	Major	Failure of the regeneration

		Minor	Partial loss of the graft

Verardi & Simion, 2007	GBR	Class I	Small soft tissue fenestration (≤ 3 mm)

		Class II	Opening wider than 3 mm

Fontana et al., 2011	GBR	Healing complications	Class I: Small membrane exposure without purulent exudate

			Class II: Large membrane exposure without purulent exudate

			Class III: Membrane exposure with purulent exudate

			Class IV: Abscess formation without membrane exposure

		Surgical complications	A: Flap damage

			B: Neurological complications

			C: Vascular complications

Jensen et al. 2016	GBR/Onlay	Soft tissue dehiscence	Separation of the suture line with exposure of the barrier membrane and/or the grafting material

		Infection	Pain, swelling, redness, fever and/or purulent discharge that require additional antibiotic treatment

		Additional augmentation procedures needed	At the time of implant placement, to obtain a sufficient implant stability and/or to create an optimal esthetic result after loss of a graft or an inadequate primary augmentation

## Data Availability

The data used to support the findings of this study are available from Professor Pietro Felice, upon request (email: pietro.felice@unibo.it).
